# Emotional State During Tasting Affects Emotional Experience Differently and Robustly for Novel and Familiar Foods

**DOI:** 10.3389/fpsyg.2020.558172

**Published:** 2020-09-25

**Authors:** Daisuke Kaneko, Anne-Marie Brouwer, Maarten Hogervorst, Alexander Toet, Victor Kallen, Jan B. F. van Erp

**Affiliations:** ^1^Kikkoman Europe R&D Laboratory B.V., Wageningen, Netherlands; ^2^Microbiology and Systems Biology, Netherlands Organization for Applied Scientific Research (TNO), Zeist, Netherlands; ^3^Perceptual and Cognitive Systems, Netherlands Organization for Applied Scientific Research (TNO), Soesterberg, Netherlands; ^4^Research Group Human Media Interaction, University of Twente, Enschede, Netherlands

**Keywords:** emotional state, novelty, memory, food pleasantness, emotion

## Abstract

Emotional state during food consumption is expected to affect food pleasantness. We hypothesize that a negative emotional state reduces food pleasantness and more so for novel foods than for familiar foods because novel foods have not yet been associated with previous emotions. Furthermore, we expect this effect to be stronger when judging the food again from memory without tasting. We induced a positive emotional state in 34 participants by telling them that they earned a monetary bonus and induced a negative emotional state in 35 other participants by subjecting them to a social stress test. After this emotion induction, both groups tasted and rated a (for them) novel soup (sumashi soup) and a familiar soup (vegetable soup). Several explicit and implicit measures of food pleasantness (rated valence, EsSense25, willingness-to-take-home and sip size) indicated that while the negative emotion group did not experience the soups as less pleasant than the positive emotion group, there was an interaction between food familiarity and emotional group. The positive emotion group experienced novel and familiar soups as equally pleasant, whereas the negative emotion group experienced the novel soup as relatively unpleasant and the familiar soup as pleasant. The latter result is consistent with a comforting effect of a familiar taste in a stressful situation. This effect remained in the ratings given 1 week later based on memory and even after retasting. Our results show that emotional state affects food pleasantness differently for novel and familiar foods and that such an effect can be robust.

## Introduction

Food judgments (as probed by, e.g., ratings of food preference or liking, food pleasantness, food choice, and eating behavior) depend not only on the quality of the taste but also on the emotional state during food consumption, the social–emotional context in which the food is consumed, and already existing associations between food and emotion (Desmet and Schifferstein, 2008; Salvy et al., 2008). Food associations are related to regional food habits, different food cultures, and food traditions in the family.

The effect of ambiance on food intake and food choice was reviewed by Stroebele and De Castro (2004). They define ambiance as a context of environmental stimuli and conclude that there are major influences of ambiance on eating behavior. The studies that they reviewed showed effects of social–emotional aspects of context, as well as effects of physical aspects of contexts [e.g., colors (Spence et al., 2010; Chen et al., 2018), sounds (Spence and Shankar, 2010; Woods et al., 2011), and/or odors (Herz et al., 2004)]. We assume that many of the reported context effects, especially the social–emotional context effects, influence food judgments through the induction of a certain emotional state. Below we review studies that examine the effect of emotional state on food experience in some more detail.

Birch et al. (1980) examined the effects of pairing positive experiences with snack foods on children’s liking of the foods. In their study, the same snack foods were served to children (1) as a reward, (2) by a friendly adult, (3) in a non-social context, or (4) at normal snack time. Children’s liking ratings were higher on snack foods in the two emotionally positive contexts (as a reward or by a friendly adult) than in the other contexts, indicating that the liking of snack foods was affected by emotion. Siegel and Risvik (1987) examined the effect of positive and negative mood on acceptance ratings of an almond dairy bar in adulthood. They induced different moods by asking participants to indicate their current state using questionnaires that contained either positively formulated statements such as “I feel great” (positive mood group) or negative statements such as “I feel weak” (negative mood group). They found that participants from the positive mood group reported significantly higher acceptance of the almond dairy bar than those in the negative group.

Kuenzel et al. (2011) aimed to induce different emotional states using video clips to investigate the effect of emotional state on food preference and liking. For 5 consecutive days, participants watched 4- to 5-min positive video clips (two different positive states: one active and one relaxed) or a neutral video clip. Two different novel uncolored drinks were developed for this study: a generally liked drink and a more neutral drink. Participants were served the liked or the neutral drinks just before the start of the film clips and were instructed to finish them by the end of the clips. The study showed an interaction between type of drink (neutral and liked) and emotional state (active, relaxed, and neutral), indicating that liking ratings of the liked drink were lower in the relaxed condition than in the neutral condition. Thus, this study shows a (modest) effect of emotional state affecting liking scores of flavored drinks, be it not simply in the direction of an emotional state that was intended to be positive leading to higher liking. The authors suggest that the reason for this effect might be that participants’ attention may have been divided when being in a positive state, leading to a tendency to score toward the middle of the liking scales, which resulted in relatively low liking scores for the liked drink.

Walsh and Kiviniemi (2014) used an “implicit priming paradigm” to create one of three emotional associations (positive, negative, or neutral) to images of fruit. This paradigm involves repeated presentation of sequential pairs of a positive, negative, or neutral image or word followed by an image of fruit. Twenty of these pairs were interspersed among a total of 230 images that were presented to each participant. At the end of the experiment, participants were asked to choose one among a selection of apples, bananas, and granola bars. Those in the positive condition were more likely to select fruit compared to those in either the neutral or negative condition.

All the studies discussed above indicate that emotional state can affect experienced food pleasantness and liking. They all used familiar foods as stimuli, except for the study by Kuenzel et al. (2011), in which only unfamiliar stimuli were used. We think that food familiarity is a key factor that may interact with emotional state when experiencing and judging food. When tasting a food for the first time (a novel food), effects of emotional state may be more pronounced than when tasting a familiar food because there is no influence yet of existing associations. Knowledge about such effects in the absence of prior existing associations is important, for instance, when introducing new products to the market or in medical settings where patients need to consume specific foods, supplements, or medicines. However, we are not aware of research exploring whether the effect of emotional state on food pleasantness indeed differs between novel and familiar food.

In addition, the majority of studies on the effect of emotional state or context focus on the instantaneous effect on food pleasantness. However, Köster and Mojet (2015) argued that the role of memory is probably much more important than the “first impression” experience that is commonly investigated. They emphasized that products should be tested for the emotions they evoke before, during, a few hours after, and a week (or even longer) after consumption, to obtain a more complete picture of the experience of the product.

In the current study, we evaluate how novel and familiar foods (two types of broths, from now on referred to as “soups”) are affected by emotional state during tasting (positive/negative), both instantaneously and a week later. We asked participants to come to the laboratory twice, separated by an interval of 1 week; we refer to the first day as Day 1, and to the second day a week later as Day 2. On Day 1, participants were asked to taste and rate a novel soup and a familiar soup. Before tasting and rating the soups, we induced a positive emotional state in half of the participants, and we induced a negative emotional state in the other half. On Day 2, participants underwent two separate sessions. In the first session, participants were asked to rate the same soups as tasted and judged on Day 1, but without tasting (i.e., from memory). In the second session, they rated the same soups again, but this time with actual tasting. The effect of emotional state on food experience of novel and familiar soups was not only measured by using self-report [valence ratings; EsSense25 questionnaire (Nestrud et al., 2016) that probes 25 emotions associated with food], but also by using behavioral measures, namely, sip size and willingness-to-take-home. These measures are of a more implicit nature, and expected to support the self-report of valence ratings (Lagast et al., 2017; Kaneko et al., 2018).

The following hypotheses are tested:

(1) Overall experienced food pleasantness, as reflected in the valence ratings and the EsSense25, is lower when tasting soups in a negative emotional state than in a positive emotional state.
(2) This effect is stronger for the novel soup than for the familiar soup.
(3) Differential effects of emotional state on novel and familiar soups will be stronger a week later when the actual taste of the soup is not available. This is because measures of experience will then only be based on memory, where the novel soup has only been associated with the experience of the (emotional) tasting session in the laboratory, and the familiar soup is also associated with other, previous food experiences.
(4) When participants subsequently taste the soups again, the effect mentioned under (3) is reduced, because experience is no longer based on memory alone.
(5) The behavioral measures of sip size and willingness-to-take-home show a similar pattern of results compared to subjective ratings.

In summary, this study will inform us about the interaction between emotional state and food familiarity on food experience, both during initial tasting and a week later.

## Materials and Methods

### Participants

A total of 70 healthy participants (19 men, 51 women) were recruited for this study. Exclusion criteria were food allergies or special diets. One of the male participants dropped out from the study. Data from this participant were excluded from all analyses, leaving us with data of 69 participants. All participants had the Dutch nationality and were between 19 and 63 years old, with an average of 48.4 years and a standard deviation of 10.4 years. Participants were recruited through the participant pool of the research institute where the study took place (TNO) and received a basic monetary reward of 30 Euros per participant to compensate for time and travel costs. On top hereof, and unknown to them beforehand, participants in the positive emotional state group received a 5 Euro bonus. Before participating in this study, all participants signed an informed consent in accordance with the Helsinki Declaration of 1975 as revised in 2014 (World Medical Association, 2014). The study was approved by the TNO Institutional Review Board. After signing the informed consent, they were randomly assigned to the positive emotional state group (34 participants: 10 men, 24 women, average age of 49.2 years) or the negative emotional state group (35 participants: 8 men, 27 women, average age of 47.7).

Sip size data were not complete for three participants (one from the positive group, two from the negative group) and were thus left out in the analysis on sip size.

We also recorded physiological data. These recordings failed for two participants (one from the positive group and one from the negative group) and were thus left out in the analyses on the physiological data.

### Materials

#### Test Stimuli

Vegetable and sumashi soup were selected as familiar and novel soups, respectively. Vegetable soup was prepared using vegetable bouillon cubes (Maggi, Nestlé, Switzerland) following the instruction on the package. Sumashi soup is a traditional Japanese transparent soup. It was prepared by mixing 5.0 g of seaweed broth (Riken Vitamin, Japan), 20.0 g of soy sauce (Kikkoman, Japan), 5.0 g of cooking sake (Wadakan, Japan), and 1.0 g of sea salt with 750 mL of hot water. The two soups were always prepared in the same way each morning and kept at approximately 60°C until they were served. Before serving the soups, a selection of regular drinks (apple juice, orange juice, yogurt drink, milk, buttermilk, rooibos tea, black tea, cola), diluted vinegar (50% vinegar, 50% water), and water were served in semirandomized order. This was done to answer other research questions (Kaneko et al., 2019). All soups were served in white plain cups, in portions of 50 g. At the end of Day 2 of the experiment, 100 g of each of the two soups was given to further assess the emotions evoked by tasting each soup.

#### Valence Scale

SAM pictures (Bradley and Lang, 1994) with nine-point scales were used for valence self-report ratings. The nine-point scale was positioned in the appropriate location at the bottom of each SAM scale, where the most leftward (most unpleasant) and the most rightward (most pleasant) parts of the scale were translated into values of 1 and 9, respectively. With respect to valence, participants were asked how pleasant their experience with the soup was, with the manikin on the right indicating a very pleasant experience and the manikin on the left a very unpleasant experience. Participants were instructed that they should try to answer quickly, without thinking too long.

#### EsSense25 Questionnaire

Besides valence scales, the EsSense25 (Nestrud et al., 2016) was used to obtain self-reported emotions evoked by experiencing the two soups. The EsSense25 is a shorter version of the EsSense Profile^§^ (King and Meiselman, 2010), which was developed to measure emotions associated with foods. Each of 25 emotional terms (*loving*, *nostalgic*, *good*, *good natured*, *joyful*, *bored*, *secure*, *happy*, *warm*, *disgusted*, *pleasant*, *active*, *satisfied*, *aggressive*, *guilty*, *calm*, *free*, *understanding*, *enthusiastic*, *interested*, *tame*, *adventurous*, *wild*, *mild*, and *worried*) was assessed on a five-point scale ranging from 1 (not at all) to 5 (very much).

#### Behavioral Measures

For the behavioral measures, sip size and willingness-to-take-home were recorded. To measure sip size, the exact weight of each soup including the cup was measured before the participant took a sip. After finishing the experiment, the cups with the remainder of each soup were weighed again to determine the sip size.

A modified rating scale of willingness-to-take-home (Wichchukit and O’Mahony, 2010) was used in this study. While in the original scale participants would be asked which soup as used in the experiment they wanted to take home as a reward, we asked participants how many cups of each soup they would want to take home after the experiment with a maximum number of 6 cups (e.g., 1 sumashi soup and 5 vegetable soup). Participants could choose fewer than 6 cups in total (e.g., none, or two sumashi soups and three vegetable soups).

#### Physiological Recording Equipment (Electrodermal Activity and Electrocardiogram)

Electrodermal activity [EDA; for skin conductance level (SCL)] and electrocardiogram [ECG; for interbeat interval (IBI), which is the inverse heart rate] were measured to assess whether the experimental induction of emotion was effective in case we would not find any effect of emotion. EDA and ECG were recorded using an Active Two MkII system (Biosemi B.V., Amsterdam, the Netherlands), with a sampling frequency of 512 Hz. SCL was measured by placing gelled electrodes on the fingertips of the index finger and the middle finger of the non-dominant hand. ECG electrodes were placed on the right clavicle and on the lowest floating left rib. SCL was measured by placing gelled electrodes on the fingertips of the index finger and the middle finger of the non-dominant hand. Electroencephalogram (EEG) was recorded as well, for different research questions (Kaneko et al., 2019).

#### Emotional State Induction

On Day 1, participants underwent either one of two types of emotional state induction, depending on the group they were assigned to. To induce a positive emotional state, participants received a message on the screen that they would receive an extra monetary bonus for participating in the experiment and that after tasting and judging the second soup they would receive the instruction to flip a card on the table that would tell them the exact amount of this bonus. This message was displayed just before displaying the name of the first soup. After tasting and judging the soups, the message to now flip the card was displayed. Participants flipped the card telling them that the amount of the bonus was 5 Euros. They received this bonus at the end of the experiment. To induce a negative emotional state, we used a modified Sing-a-Song Stress Test, which has been shown to induce profound social stress (Brouwer and Hogervorst, 2014; Brouwer et al., 2017; Toet et al., 2017). Just before the first soup, a message was displayed that they would receive the instruction to sing a song out loud after tasting and judging the second soup. This instruction was given as announced, and participants started singing a song. The aim of these emotion induction procedures was to induce emotions that were as different as possible with respect to pleasantness in the two groups, while keeping other elements (such as receiving an announcement about an exciting task to perform after tasting) as similar as possible.

### Experimental Design and Procedure

Participants came to the laboratory twice. There were minimally 5 days and maximally 8 days (on average 6.97 days) between the first (Day 1) and the second (Day 2) recording session.

#### General Procedure Day 1

**FIGURE 1:**
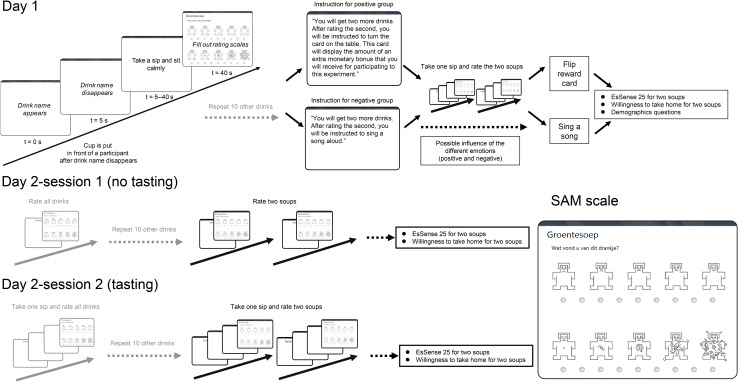
Schematic overview of an experimental trial and of the experimental procedure in Day 1, Day 2–1, and Day 2–2. At the bottom-right, a rating screen is depicted, showing the small circles below the SAM scales that participants clicked in order to give their responses, upon which a new trial started. In Day 1, participants are separated into two groups, with either positive or negative emotion induction. Emotion induction occurred before tasting and rating two soups. In Day 2–1 and Day 2–2, all participants followed the exact same procedure. Parts of the procedure highlighted in gray served to answer other research questions (Kaneko et al., 2019).

After participants arrived at the laboratory, the experimental procedure was explained by the experimenter. They were informed that they were going to take part in a study on food experience, in which they would taste and judge drinks and soups. Participants were not told about the emotion induction. After the explanation of the study, participants signed the informed consent and, unknown to them, were randomly assigned to the positive or negative emotional state group. Electrodes for measuring EDA, ECG, and EEG were attached, and participants were asked to sit comfortably in front of a computer screen. Participants were instructed how to take one sip and practiced an experimental trial. At this time, when the participant was in the negative emotional group, one of experimental leaders came in, pretending to be a next participant who arrived at the laboratory earlier than the appointed time. The other experimental leader asked the fake participant to stay in the same laboratory room to wait for the previous participant to finish the experiment. Thus, the fake participant was in the room during the whole experiment for the negative emotional state group. In the positive emotional state group, only the experimenter was present. Participants filled out a general questionnaire on demographic details and current emotional state. A tasting and rating trial (schematically depicted in the top left of Figure 1) went as follows. First, the name of the test sample was presented on the screen. This was the sign for the experimenters to place the appropriate cup in front of the participant. After 5 s, the name of the test sample disappeared, which was the sign for the participant to take one sip. After taking the sip, participants sat still and looked at a blank white screen. Forty seconds after the name had appeared on the screen, the self-report SAM rating scales appeared. After entering the scores, the next trial started. This procedure was repeated until all drinks had been served (depicted in gray in Figure 1). Immediately after, the group-dependent emotional state was evoked through an instruction screen as outlined above. Then, participants in both groups were served the two soups following the same procedure as before and after rating the second soup performed the task as instructed (i.e., either flip the reward card or sing a song). Sumashi soup was presented as “Asian soup” to participants. Half of the participants first tasted the vegetable soup and half the sumashi soup. After participants completed the task, another 100 g of the two soups was served to all participants in the same serving order as they had tasted and rated before. This time participants could taste more than once and were asked to more elaborately self-report their emotions evoked by tasting each soup using the EsSense25. After filling out the EsSense25 questionnaires, participants were asked to answer whether they were familiar with the taste of “Asian soup” and to write down the name of the soup if they knew the name or wanted to make a guess. In the end of the experiment, we asked participants how many cups of vegetable soup and Asian soup they would want to bring home if they would receive them for free (with a maximum of 6 in total). They did not actually receive such cups of each soup to prevent them to consume the soups (more than they usually would do) the days preceding Day 2.

#### General Procedure Day 2

The session on Day 2 was divided into two parts and was conducted without any physiological measures and without emotion induction. The schematic experimental procedure is summarized in the bottom half of Figure 1. First, participants were asked to sit in front of a screen and rate each drink and soup without tasting them, only relying on their memorized experience from 1 week ago. The name of the drink or soup appeared on the screen, followed by the SAM scales as on Day 1, but without the 40-s blank screen period in between. For each participant, the order of the drinks and soups was the same as on Day 1. Next, participants were asked to rate their emotions with the two soups using the EsSense25, i.e., based only on their memory of the taste and the emotions they had encountered a week before. Then, they were asked again for the two soups they would want to take home in the same manner as on Day 1. After this first session of Day 2 (referred to as Day 2–1), the second session of Day 2 (Day 2–2) commenced. In this second session, the same procedure was repeated, but this time with tasting and rating the drinks and soups in exactly the same manner as on Day 1. This also included judging the soups using the EsSense25, while being provided with 100 g of each of the two soups. Finally, as on Day 1, we asked how many cups of vegetable soup and Asian soup they would like to take home. After they completed the experimental tasks in Day 2, the experimenters debriefed participants on the purpose of the study and the emotion induction procedures.

### Data Processing and Statistical Analysis

#### Main Dependent Variables

Our main dependent variables reflecting food experience are valence ratings, EsSense25 ratings, sip size, and willingness-to-take-home.

Statistical analyses on dependent variables were conducted using SPSS version 25. To investigate the main effects and interactions of soup (familiar vegetable soup and novel sumashi soup), session (Day 1, Day 2–1, and Day 2–2), and emotional state (positive and negative), we performed mixed model analyses (maximum likelihood approach) with soup (2) and session (3) as within-subjects variables, and state (2) as between-subjects variable. For sip size, session involved two rather than three levels (Day 1 and Day 2–2) because participants did not take a sip in the Day 2–1 session.

For all statistical tests, we considered an α level of 0.05. Given that the EsSense25 features 25 variables, correction for multiple testing is in place. Therefore we also interpret these results within the light of the Bonferroni-corrected α level of 0.002. Least significant difference (LSD) *post-hoc* comparisons were performed to interpret any significant interactions that, in the case of EsSense25, survive the Bonferroni correction. This came down to *post-hoc* comparisons that elucidated state × soup interactions in six measures.

### SCL and IBI

Custom-made MATLAB 2019a^1^ algorithms were used to extract SCL and IBI. To examine the effect of the instruction that was intended to induce either positive or negative emotion on SCL and IBI, the following steps were followed. First, the EDA signal was bandpass filtered between 0.03 and 100 Hz. IBI, defined as the temporal distance between R-spike (Appelhans and Luecken, 2006), was extracted from the ECG signal using custom-made algorithms. Next, for each participant, EDA was averaged across the 40 s starting at onset of the announcement of the first soup that was presented immediately after the message that induced the positive or negative emotion. The same was done for the last drink that was presented before the message that induced the positive or negative emotion. This latter value served as a baseline. After log transforming the values, the baseline was subtracted from the value obtained after the emotion induction. The same procedure was followed for IBI. An increase in emotional arousal would be reflected by decreased IBI (i.e., increased heart rate) and increased SCL (Brouwer and Hogervorst, 2014). We examined whether these differential values were indeed statistically different from zero using one-sample *t*-tests. We also compared them between the positive and negative emotional state groups by using two-sample *t*-test.

## Results

### Verifying the Experimental Manipulations

#### Emotion Induction

**FIGURE 2:**
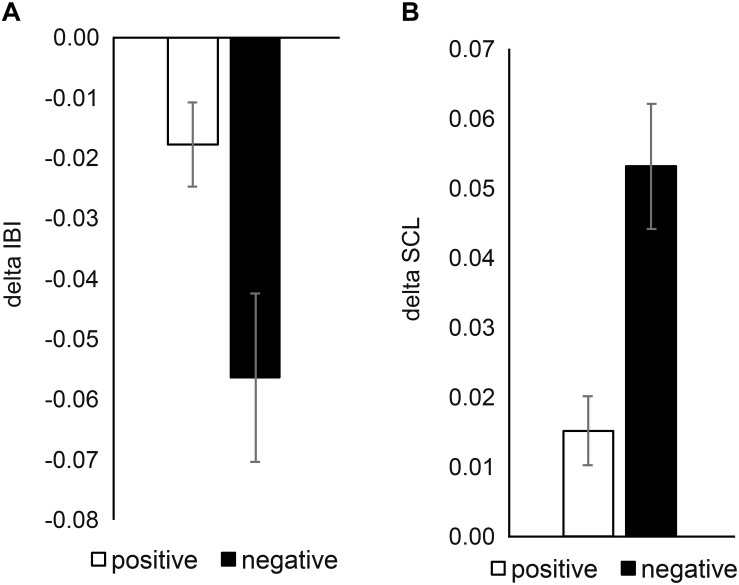
Mean delta values for **(A)** IBI and **(B)** SCL between before and after announcement in positive emotional group and in negative emotional group. Error bars indicate standard error of the mean.

Figure 2 shows the average difference of the mean SCL and the mean IBI before and after the positive emotion induction (announcement to flip a bonus reward card after tasting and rating two soups) and the negative emotion induction (announcement to sing a song after tasting and rating two soups). As expected, IBI decreased and SCL increased for both positive emotion induction [IBI: *t*(32) = -2.61, *p* = 0.014; SCL: *t*(33) = 2.89, *p* = 0.007] and for negative emotion induction [IBI: *t*(32) = -4.14, *p* < 0.001; SCL: *t*(33) = 6.09, *p* < 0.001]. This shows that both types of emotion inductions indeed elicited arousal. Two-sample *t*-tests indicated that elicited emotional arousal was even stronger for the negative emotion induction than for the positive emotion induction [IBI: *t*(65) = 2.51, *p* = 0.015; SCL: *t*(65) = -3.70, *p* < 0.001].

#### Novelty of Foods

None of the participants reported to have experienced the taste of sumashi soup before, and none of them were able to answer the question of what type of “Asian soup” had been used in this study, indicating that the sumashi soup can indeed be considered as a novel soup for all participants in this study.

### Effect of Emotional State

Table 1 presents the results of the mixed-model analyses for each of the dependent variables. Significant effects are marked in light gray. Table 2 presents *post-hoc* comparisons that elucidate significant soup × state interactions. In the following sections, we focus on the statistical results that are directly connected to our hypotheses.

**TABLE 1:** Summary of the statistical data obtained with a mixed-model analysis for each of the dependent variables.

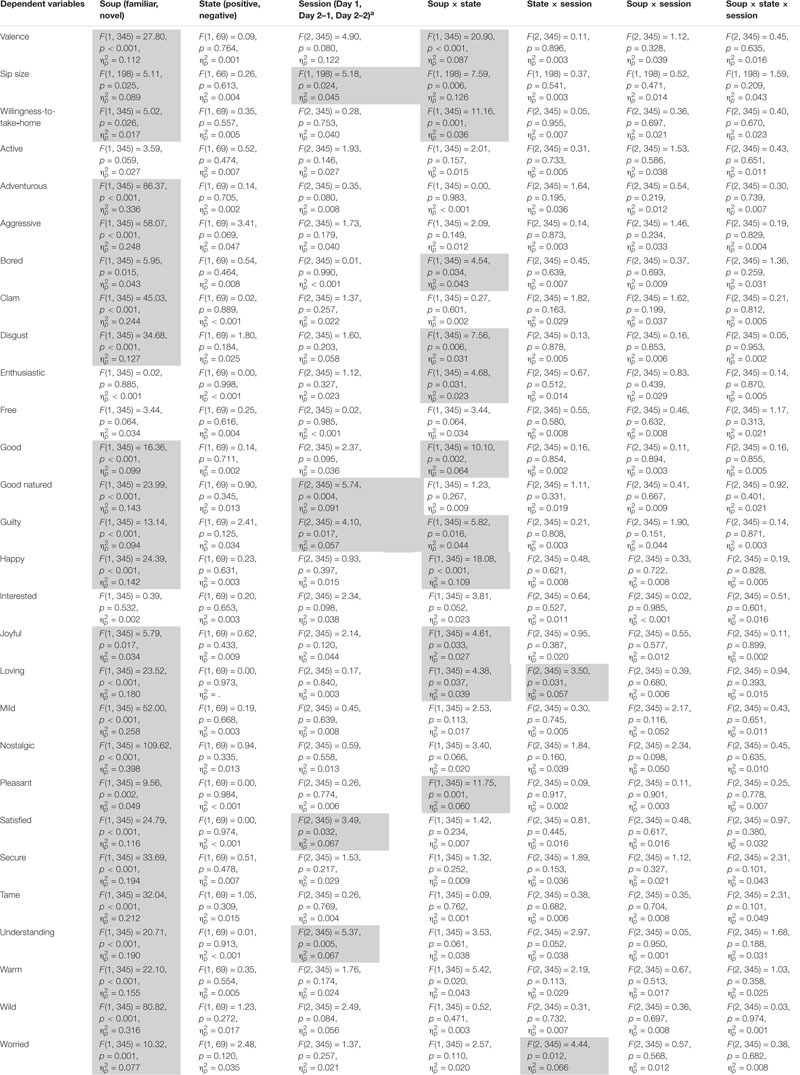

**The significant main effects and interactions were highlighted in light gray. Variables below the dashed lines reflect the EsSence25 variables. ^*a*^There are only two levels of session for sip size (Day 1 and Day 2–2).**

**TABLE 2:** Summary of LSD *post-hoc* comparisons that elucidate significant soup × state interactions.

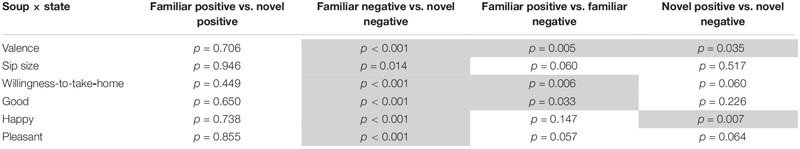

**The significant effects are highlighted in light gray. Variables below the dashed lines reflect the EsSence25 variables.**

#### Valence Ratings

**FIGURE 3:**
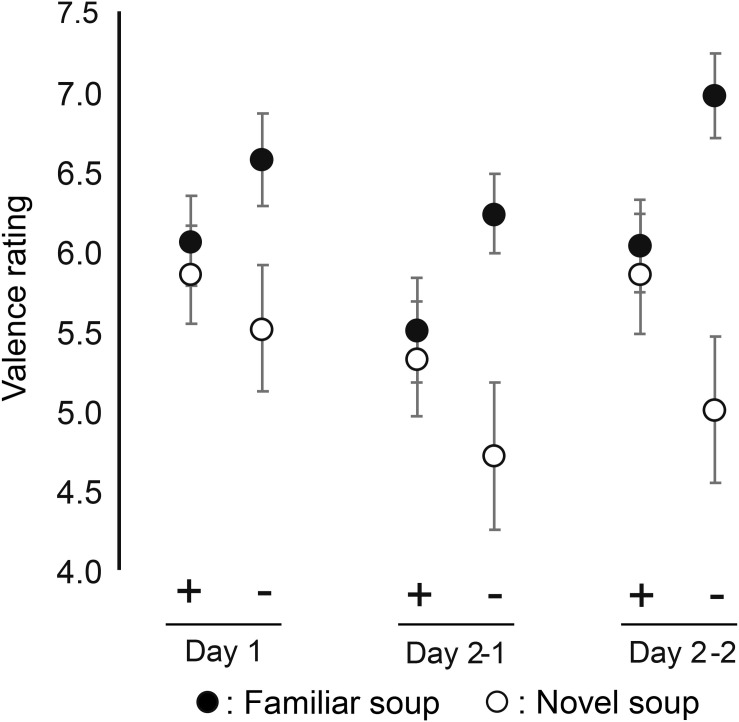
Mean valence ratings of familiar and novel soup by positive emotional group (+) and negative emotional group (−) in Day 1, Day 2–1, and Day 2–2. Error bars indicate standard error of the mean.

Reported mean valence of each soup averaged across participants for each of the three sessions (Day1, Day2–1, and Day 2–2), each of the two emotional states (positive/negative), and each of the two soups (familiar/novel) is presented in Figure 3. There was no main effect of emotional state on valence ratings (Hypothesis 1) [*F*(1, 69) = 0.09, *p* = 0.764, η${}_{p}^{2}$ = 0.001], but we found a significant interaction effect between emotional states and soups [*F*(1, 345) = 20.90, *p* < 0.001, η${}_{p}^{2}$ = 0.087]. The *post-hoc* tests indicated that the novel soup was judged as less pleasant than the familiar soup in the negative emotional state, whereas there was no difference between the ratings of the two soups in the positive emotional state (first two columns in Table 2). *Post-hoc* tests also indicated that the familiar soup was judged as more pleasant in the negative than in the positive emotional state and that the novel soup was judged as less pleasant in the negative than in the positive emotional state (last two columns in Table 2) (Hypothesis 2). A lack of interaction between state, soup, and session indicates that this effect remains constant across sessions (Hypotheses 3 and 4).

#### Self-Reported Emotions (EsSense25)

**FIGURE 4:**
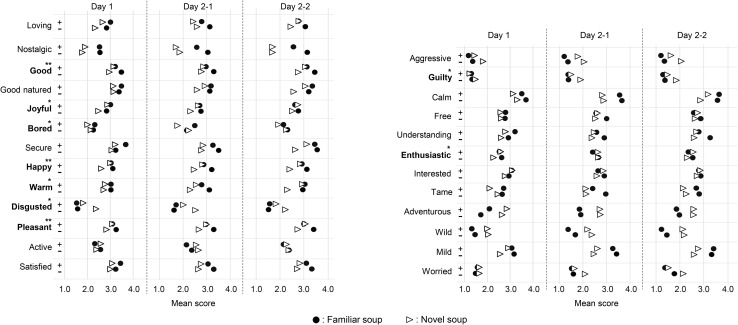
Mean rated scores of each emotion based on EsSense25 of familiar and novel soup by positive emotional group (+) and negative emotional group (−) in Day 1, Day 2–1, and Day 2–2. Bolded emotions indicate significant interactions between emotional states and soups with *p < 0.05 and with **Bonferroni correction of *p* < 0.002.

Figure 4 shows the mean ratings for the 25 emotions of each soup averaged across participants for each of the three sessions (Day1, Day2–1, and Day 2–2) in each of positive and negative emotional state. As can be seen in Table 1, similar to what was found for valence ratings, there was no main effect of emotion (Hypothesis 1), whereas for nine out of 25 emotions the analyses revealed significant interactions between emotional states and soups (Hypothesis 2). Six of these nine emotions concerned positive emotions [*happy*, *F*(1, 345) = 18.80, *p* < 0.001, η${}_{p}^{2}$ = 0.109; *pleasant*, *F*(1, 345) = 11.75, *p* = 0.001, η${}_{p}^{2}$ = 0.060; *good*, *F*(1, 345) = 10.10, *p* = 0.002, η${}_{p}^{2}$ = 0.064; *warm*, *F*(1, 345) = 5.42, *p* = 0.020, η${}_{p}^{2}$ = 0.043; *enthusiastic*, *F*(1, 345) = 4.68, *p* = 0.031, η${}_{p}^{2}$ = 0.023; *joyful*, *F*(1, 345) = 4.61, *p* = 0.033, η${}_{p}^{2}$ = 0.027]. They all showed the same pattern as rated valence, namely, stronger positive emotions for the familiar soup than the novel soup, for negative compared to positive emotional state. Note that only *happy*, *pleasant*, and *good* pass the Bonferroni-corrected α level of *p* = 0.002. As for valence, *post-hoc* comparisons indicate that there was no significant difference between the soups in the positive emotional state, but that in the negative emotional state, familiar soup was more positively judged than the novel soup. The negative emotions, *disgusted* and *guilty*, which showed a significant interaction between soup and state, revealed a consistent pattern with stronger-rated negative emotions for the novel soup than the familiar soup, for negative compared to positive emotional state [*disgusted*, *F*(1, 345) = 7.56, *p* = 0.006, η${}_{p}^{2}$ = 0.031; *guilty*, *F*(1, 345) = 5.82, *p* = 0.016, η${}_{p}^{2}$ = 0.027]. Finally, *bored* showed a significant soup × state interaction [*F*(1, 345) = 4.54, *p* = 0.034, η${}_{p}^{2}$ = 0.043], indicating that participants with a positive emotional state rated the novel soup as less boring than the familiar soup for the positive rather than the negative emotional state, fitting with the other EsSense25 and valence results. However, none of the effects found for negative emotions pass the Bonferroni-corrected α level. For none of the emotions was a significant three-way interaction found, indicating that the interaction effects between soup and emotional state are stable across sessions (Hypotheses 3 and 4).

#### Behavioral Measures: Sip Size and Willingness-to-Take Home

**FIGURE 5:**
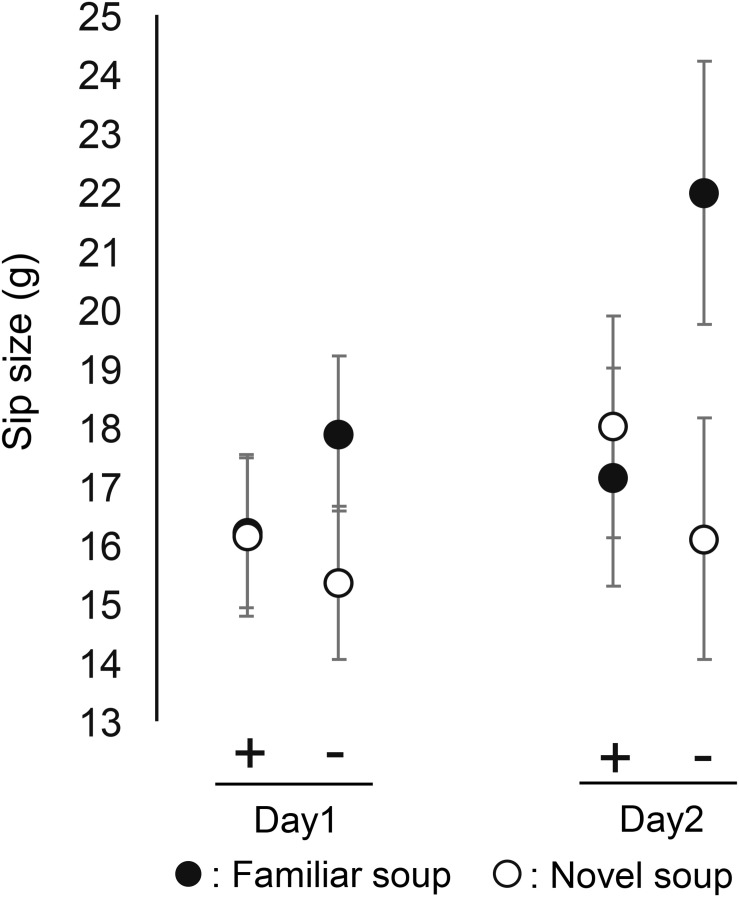
Mean sip size of familiar and novel soup by positive emotional group (+) and negative emotional group (−) in Day 1 and Day 2–2. Error bars indicate standard error of the mean.

**FIGURE 6:**
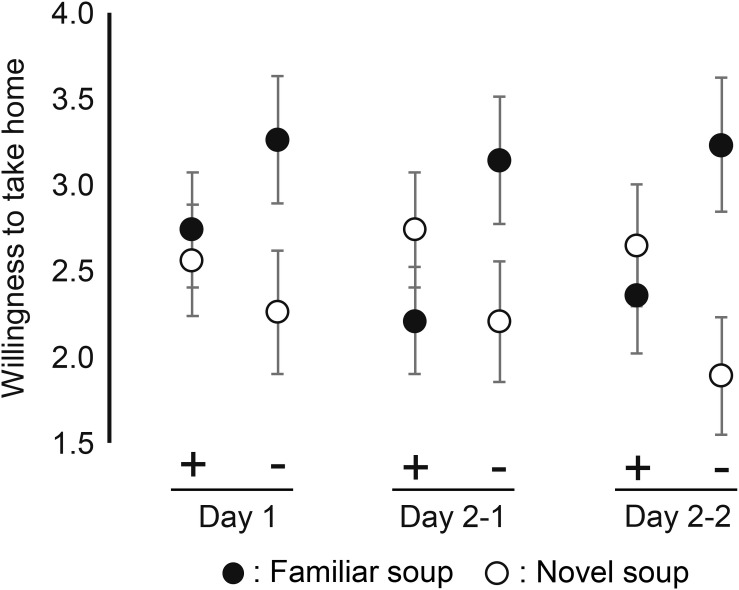
Mean willingness-to-take-home of familiar and novel soup by positive emotional group (+) and negative emotional group (−) in Day 1, Day 2–1, and Day 2–2. Error bars indicate standard error of the mean.

Figure 5 shows the mean sip size for each soup, each of the two sessions that included sip size (Day1 and Day2–2, not Day 2–1), and each emotional state. Figure 6 shows the mean number of cups of soup participants would want to take home (willingness-to-take-home) averaged across participants for each of the three sessions and of two emotional states. These behavioral measures showed a similar pattern of effects as the subjective ratings. There was no main effect of emotional state on sip size [*F*(1, 66) = 0.26, *p* = 0.613, η${}_{p}^{2}$ = 0.004] and willingness-to-take-home [*F*(1, 69) = 0.35, *p* = 0.557, η${}_{p}^{2}$ = 0.005], but significant interactions between emotional state and soups on both sip size [*F*(1, 198) = 7.59, *p* = 0.006, η${}_{p}^{2}$ = 0.126] and willingness-to-take-home [*F*(1, 345) = 11.16, *p* = 0.001, η${}_{p}^{2}$ = 0.036]. Similar to valence and EsSense25 ratings, sip size and willingness-to-take-home were lower for novel soup than for familiar soup in the negative emotional state, while there was no difference between soups in the positive emotional state. This was corroborated by *post-hoc* comparisons. No significant three-way interactions were found for both measures, indicating a stable effect of emotion on familiarity across sessions. Table 1 shows that also the main effect of soup and the lack of effect of state × session that were observed for valence ratings, and most of the Essense25 ratings are mirrored in the patterns of sip size and willingness-to-take-home (Hypothesis 5).

## Discussion

The present study investigated the effect of emotional state (positive and negative) on valence and EsSense25 ratings, reported willingness-to-take-home, and sip size for novel and familiar soups, both at the time of emotion induction (Day 1) as well as at two recording sessions a week after (Day 2–1, without tasting, and Day 2–2, with tasting).

At Day 1, participants tasted and rated the soups for the first time, after an either positive or negative emotion induction procedure. For Day 1, we expected that overall experienced food pleasantness, as reflected in the valence ratings and the EsSense25, would be lower when tasting soups in a negative emotional state than in a positive emotional state and that this effect would be stronger for the novel soup than for the familiar soup. We indeed observed that participants with a negative emotional state rated lower valence for the novel soup than for the familiar soup, whereas there was no difference between soups in the positive condition. However, the lack of a main effect of emotional state indicated that this was not merely due to a general lower valence in the negative condition. Rather, familiar soup was rated more positively in the negative emotional condition than in the positive emotional condition. For EsSense25, this pattern was found for three positive emotions (*happy*, *pleasant*, and *good*) and an additional three when a more liberal criterion of significance was taken (*warm*, *joyful*, and *enthusiastic*) and three negative emotional terms (only without Bonferoni correction: *bored*, *disgusted*, and *guilty*). These results force us to reject Hypothesis 1—we did not find that negative emotional state decreased experienced food pleasantness in general, but partly supported Hypothesis 2—we found that negative emotional state decreased food pleasantness particularly for novel foods, where familiar food, contrary to our expectation, rather increased in food pleasantness. Our results are consistent with a comforting effect of a familiar taste in a stressful situation.

In the negative emotional group, we found that familiar soup was preferred over novel soup. In general, familiar foods are reported to be preferred over unfamiliar food. Fenko et al. (2015) investigated participants’ hedonic responses to various familiar and unfamiliar soy product images and found higher liking scores for familiar products, as well as a more positive expectation of the familiar products’ taste. Consistent with this, Toet et al. (2019) found that Asian and Western participants rated food from their own culture as more positive. Our study shows that this tendency may be especially strong in stressful situations. This is also suggested by a study from Locher et al. (2005). They asked participants to bring foods that “made them feel good” or “provided them comfort” and to explain why this was so. They concluded that people consume familiar foods to relieve feelings of distress and anxiety and that novel foods cannot fulfill this need because they tend to evoke more feelings of anxiety. Other studies report that individuals in depressed moods show a preference for and consume palatable well-known “comfort foods” to alleviate their negative feelings (Macht, 2008; Singh, 2014). Also, it is reported in a review that familiar foods represent the sense of perceived “comfort,” while it is absent with novel foods because of a lack of knowledge of them (Aldridge et al., 2009).

We hypothesized that the differential effects of emotional state on novel and familiar soups would be stronger a week later when the actual taste of the soup is not available (Hypothesis 3). This pattern could be observed in most measures. For valence ratings, the difference between familiar and novel soup in Day 2–1 tended to be larger for participants that had been under negative stress than for participants from the positive emotional condition. The EsSense25 measures showed similar effects for certain positive emotions, such as “*good*,” “*joyful*,” “*happy*,” “*warm*,” “*pleasant*,” and “*enthusiastic*,” as well as sip size. However, significant three-way interactions between soup, state, and session were far from significant for any of the measures. Thus, we conclude that the differential effect of emotion on experiencing familiar and novel soup on Day 1, when the emotions were induced, remained the same a week later, therewith rejecting Hypothesis 3.

We expected that when participants would taste the soups again (Day2–2), this would reduce the effects of memory (Hypothesis 4). However, the lack of three-way interactions between soup, state, and session showed this not to be the case—even after tasting the soups again, the interactive effect of soup and emotional state remained the same for all measures. Hypothesis 4 was therefore rejected. Our data showed that the interactive effect of emotional state and familiarity (soup) is robust. For ratings, this may have been caused by participants being inclined to give similar answers as they did before. De Wijk et al. (2019) pointed out that memories of previous encounters with the same test food may induce the use of similar ratings in new encounters. However, the finding that our implicit measure of sip size produced the same results argues against this explanation in our study.

We used different measures to evaluate food experience from various angles. Valence rating, sip size, and willingness-to-take-home showed similar patterns of effects of soup, state, and session. This pattern was also seen in emotions probed in the EsSense25. Our Hypothesis 5 was thus confirmed.

Overall, we found that the results related to participants’ food evaluations (valence, EsSense25, willingness-to-take-home, and sip size) did not completely follow our hypotheses. In fact, no main effects were observed of emotional state, although we did find significant interactions between emotional state and food novelty in all measures. The results showed that in the negative emotional condition, familiar foods were rated more positively than novel foods, whereas they were rated the same in a positive emotional state. We had expected perception of familiar foods to be more stable than novel foods across emotional conditions because of a long-term emotional association in memory in the participants. The fact that a food is familiar can be taken to mean that it is “safe,” and thus in general more positive than novel foods. In the negative, stressed condition, individuals may have been more sensitive to any potential threats, resulting in an increased avoidance (or negative valence). On the other hand, when in a positive state, there is no reason to activate the threat awareness or avoidance mechanism, and individuals do not avoid food just because it is unfamiliar. The ratings in the positive emotional group may be mainly based on the smell and taste and not so much affected by the fact that they have not experienced it before. For future studies, it would be of interest to investigate if and how food neophobia affects these interactions between emotional state and food novelty on experienced food pleasantness.

The results in this study implicate that one should introduce a new product (or a novel food) in a situation where people are not stressed. Introducing a new product to consumers who are likely to be stressed (e.g., in a hospital) would not be recommended as it may affect food pleasantness negatively and for a long period. Once people have experienced a new product in a stressed state, the negative effect is robust at least for 1 week. Therefore, a positive recommendation would be to let people taste a new product when they are in a positive mood, e.g., at a festival or after a happy movie.

## Conclusion

This study evaluated the interaction effects between emotional states and food novelty on food experiences in terms of valence and EsSense25 ratings, willingness-to-take-home, and sip size, both during initial tasting of two soups and a week later. We showed that the emotional state affected all these measures in a similar way, with low experienced pleasantness for novel food and high pleasantness for familiar food in the negative compared to the positive emotional group, in which no differences in pleasantness were found. Also, the effects of emotional state proved to be robust over time (1 week later in this study). Our findings in this study provided relevant insights for food industries and restaurants for introducing their new products to consumers and for hospitals and care institutions for providing medication or food supplements.

## Data Availability Statement

The raw data supporting the conclusions of this article will be made available by the authors, without undue reservation, to any qualified researcher.

## Ethics Statement

The studies involving human participants were reviewed and approved by the TNO Institutional Review Board (TCPE). The patients/participants provided their written informed consent to participate in this study.

## Author Contributions

DK, A-MB, VK, and JE: conceptualization. DK and MH: data curation and software. DK, A-MB, MH, and JE: formal analysis. DK: funding acquisition, resources, and writing—original draft. DK, A-MB, MH, AT, VK, and JE: investigation. DK and A-MB: methodology, project administration, validation, and visualization. A-MB, AT, VK, and JE: supervision and writing—review and editing. All authors contributed to the article and approved the submitted version.

## Conflict of Interest

DK was employed by company Kikkoman Europe R&D Laboratory B.V. The remaining authors declare that the research was conducted in the absence of any commercial or financial relationships that could be construed as a potential conflict of interest.

## Footnotes

1 www.mathworks.com
